# Biosynthesis of luminescent CdS quantum dots using plant hairy root culture

**DOI:** 10.1186/1556-276X-9-686

**Published:** 2014-12-18

**Authors:** Mariya N Borovaya, Antonina P Naumenko, Nadia A Matvieieva, Yaroslav B Blume, Alla I Yemets

**Affiliations:** Department of Genomics and Molecular Biotechnology, Institute of Food Biotechnology and Genomics, Natl. Acad. of Sci. of Ukraine, Osypovskogo Str., 2a, Kiev, 04123 Ukraine; Department of Physics, Taras Shevchenko National University, Prospect acad. Glushkova, 4, Kiev, 03022 Ukraine; Institute of Cell Biology and Genetic Engineering, Natl. Acad. of Sci. of Ukraine, acad. Zabolotnogo str. 148, Kiev, 03680 Ukraine

**Keywords:** Biosynthesis, CdS quantum dots, Luminescent, Hairy root extract, Transmission electron microscopy

## Abstract

**Abstract:**

CdS nanoparticles have a great potential for application in chemical research, bioscience and medicine. The aim of this study was to develop an efficient and environmentally-friendly method of plant-based biosynthesis of CdS quantum dots using hairy root culture of *Linaria maroccana* L. By incubating *Linaria* root extract with inorganic cadmium sulfate and sodium sulfide we synthesized stable luminescent CdS nanocrystals with absorption peaks for UV-visible spectrometry at 362 nm, 398 nm and 464 nm, and luminescent peaks at 425, 462, 500 nm. Transmission electron microscopy of produced quantum dots revealed their spherical shape with a size predominantly from 5 to 7 nm. Electron diffraction pattern confirmed the wurtzite crystalline structure of synthesized cadmium sulfide quantum dots. These results describe the first successful attempt of quantum dots synthesis using plant extract.

**PACS:**

81.07.Ta; 81.16.-c; 81.16.Rf

## Background

Nanometer-sized binary chalcogenides belonging to the groups II–VI semiconductors such as CdS, PbS, ZnS, CdSe, PbSe, etc. have attracted considerable attention due to their unique if compared to their bulk counterparts properties related to the size quantization effects [1]. Many physical properties of nanostructured semiconductors depend strongly on their size, shape and crystal structure. Thus, the development of well-controlled synthetic methods for tailoring nanocrystal shape and elucidation of the mechanisms by which the size and shape of the nanocrystals can be controlled are important issues in nanomaterials chemistry [2].

For example, CdS has a Bohr radius of 2.4 nm^2^ and direct band gap of 2.4 eV^3^ and is used in photovoltaics, in light-emitting diodes for flat-panel displays, and in other optical devices based on its nonlinear properties [3]. The lifetime of the lowest allowed optical excitation ranges from tens of a picosecond to several nanoseconds [4]. CdS has been extensively studied recently [4, 5]. Moreover, such quantum dots (QDs) have significant advantages in chemical and biological researches in contrast to traditional fluorescent organic dyes and green fluorescent proteins due to photobleaching, low signal intensity and spectral overlapping of the latter [6]. These properties of CdS QDs have attracted great interest in biology and medicine in recent years. Currently QDs are considered as potential candidates for luminescent probing and labeling in biological applications ranging from molecular histopathology and disease diagnostics to biological imaging [7].

However, the problem concerning potential toxicity of II-IV QDs (such as CdS or CdSe) has cast doubts on their practical use in medicine. A critical factor that determines the cytotoxicity of QDs is the leakage of toxic metal ions from the core caused by photolysis and oxidation [7, 8]. One promising solution to this problem is that QDs can be capped with chemically inert materials such as silica to protect the cell avoiding both cell cytotoxicity and chemical or metabolic degradation of the QD labeling inside the cells [9]. In addition, for biological studies QDs should be water soluble in order to adapt the biological environment [7].

The preparation of CdS QDs has been carried out using various methods such as microwave heating, microemulsion synthesis and ultrasonic irradiation [1, 10, 11]. However chemical methods are complicated, outdated, costly, inefficient, have low productivity and produce hazardous toxic wastes raising environmental safety and human health concerns. Therefore alternative approach suggests the use of the biological systems for synthesis of nanomaterials in order to produce nanoparticles at ambient temperature and pressure without requiring hazardous agents and generating poisonous by-products [12]. Living organisms show a unique potential in environmentally friendly production and accumulation of nanoparticles with different shapes and sizes. Studying the biological mechanisms and enzymatic processes of nanoparticle production the researchers have been focusing mainly on microorganisms including yeasts and fungi [13–16].

Meanwhile it was recently found that plants have several advantages for nanoparticles production because they are easily available, safe to handle and contain a broad range of biomolecules such as alkaloids, terpenoids, phenols, flavanoids, tannins, quinines etc. known to mediate synthesis of nanoparticles [17]. A comparatively new and unexplored technique is a biosynthesis of cadmium sulfide nanocrystals using plant extracts. Very little is known about the interaction between plant biomolecules and cadmium ions. Consequently, it is necessary to understand biosynthetic mechanism involved in the plant-based fabrication of stable CdS nanoparticles.

Therefore, the purpose of this study was to develop green, fast and easy reproducible approach for biological synthesis of CdS nanoparticles by hairy root extract of *Linaria maroccana* and characterize their structural, morphological and optical features. As a result CdS quantum dots with a spherical shape and a size from 5 to 7 nm predominantly were obtained. Synthesized CdS nanocrystals have typical for quantum dots optical properties such as specific absorption and luminescent spectra. Their main luminescent peaks corresponded to 425, 462, 500 nm.

## Methods

Biological synthesis of CdS nanoparticles was performed using hairy root culture of *L. maroccana* L. Hairy root culture was obtained using *Agrobacterium*-mediated transformation as described by us early [18]. Plant culture was grown in a liquid hormone free MS medium [19] at 28°C during 10 days. Then the resulting hairy root culture was washed five times in sterile distilled water in order to eliminate the residual culture medium. Subsequently, roots were cut with a scalpel into small pieces with a size around 2–3 mm and heated to 60°C. After that an aqueous extract of the *Linaria* hairy roots was filtered through filter paper to remove the tissue residues. Freshly obtained extract was cooled down at a room temperature and immediately used for the biosynthesis of cadmium sulfide nanoparticles.

### Biosynthesis of CdS quantum dots

In order to produce cadmium sulfide quantum dots 2 ml of 0.025 М CdSO_4_ solution (Sigma-Aldrich, USA, 99.99% purity) was poured into 100 ml flask with 30 ml of a hairy root aqueous extract of *L. maroccana* and incubated for 3 days at 28°C in the thermostat. Then 500 μl of 0.5 М Na_2_S solution (Sigma-Aldrich, USA, 98% purity) was added to the flasks which were incubated for 4 days at 28°C to produce a homogeneous clear solution of CdS nanoparticles with bright yellow color. Prior to further physical analysis of CdS nanoparticles the samples of nanoparticles solution (2 mL) were collected and centrifuged at 5000 rpm for 10 min (MiniSpin Eppendorf, USA) The solution without added CdSO_4_ was used as a control.

### UV-Visible spectrophotometry

CdS nanoparticles absorption spectra were measured using a spectrophotometer Specord UV–VIS Analytik Jena AG (Germany). Recording the absorption spectra of samples was carried out in standard quartz 10 mm-cuvettes (transmission range 170÷1000 nm). According to the protocol the accuracy of recording the wave numbers was 20 cm ^-1^ according. However due digital processing and random factors the actual experimental accuracy was 80 cm^−1^. The optical density was determined with an accuracy of up to 1% of the length of the optical scale in the optical density range from 0 to 1.4. The spectrum which was recorded by a chart recorder Specord UV VIS was analyzed by a computer scanner and converted into the figure as a jpeg file. Then the resulting file was processed by a software package GetData converting the spectrum data into a numerical format of dat-file. The numerical data were processed by software Origin Pro 8.0. To determine the wavelength corresponding to the centers of the absorption maxima each spectrum was separated into three components. Their shape was described by the Lorentz function. By varying the peak intensity, width and position of these components were replaceable. Such spectral division was carried out using automatic software for spectroscopy PeakFit 4.11.

### Luminescence of CdS quantum dots

Luminescence spectra were measured at room temperature using the serial spectrophotometer Cary Eclipse (Varian Inc., Agilent Tech. USA). The highest resolution of this spectrophotometer was 1.5 nm and was determined by the apparatus function and the smallest width of a gap. Selected spectral gap width for the measurement was 5 nm. Accuracy of recording the wavelength was 0.05 nm and the accuracy of determining the intensity did not exceed 1%. A software of the device provided an opportunity to correct the spectra by taking into account the sensitivity curve considering the spectral sensitivity of multiplier photocell used in a fluorimeter, Standard quartz cuvettes (1 × 1 × 3 cm^3^) were used for spectral measurements. For correct determination of the wavelengths spectral array was separated into three components. Their shape was described by the Lorentz function. Such spectral division was carried out using an automatic software for spectroscopy PeakFit 4.11.

### Transmission electron microscopy (TEM)

Characterization of CdS quantum dots was performed using electron microscope JEOL JEM-2100 F (Japan) with accelerating voltage 200 kV. Each sample was dispersed ultrasonically to separate individual particles, and some drops of the suspension were deposited onto carbon coated copper grids. Experimental material was precipitated by evaporation and used for further studies.

### Electron diffraction analysis

Electron diffraction patterns for the CdS nanocrystals deposited on the carbon coated copper grid were obtained using electron microscope JEOL JEM-2100 F (Japan) at electron beam energy *E* = 200 kеV (wavelength of electrons *λ* = 0, 27 nm). Localization of the beam on the sample was 200 nm.

## Results and discussion

**Figure 1:**
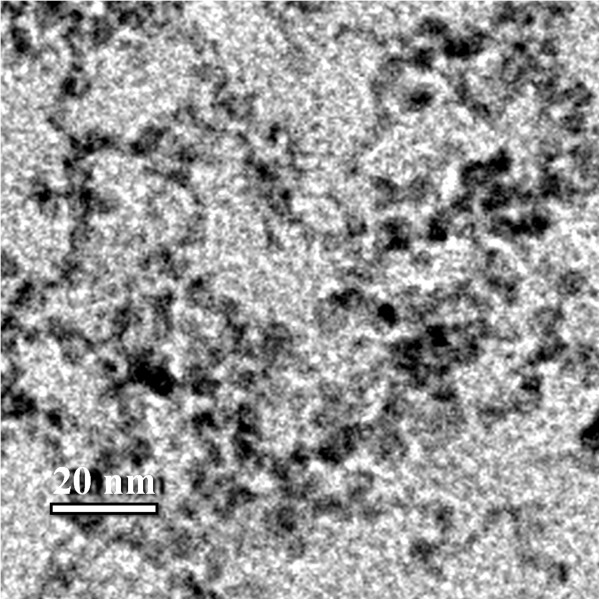
**TEM micrographs of individual CdS nanoparticles.** Synthesized samples were dispersed ultrasonically to separate individual particles, and some drops of the colloidal suspension were deposited onto carbon coated copper grids. Scale bar indicates 20 nm.

**Figure 2:**
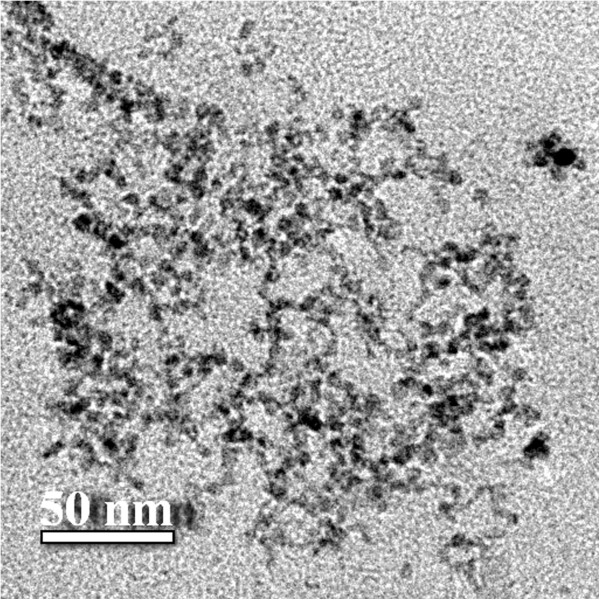
**Nanoparticles had mostly a spherical shape, size in the range from 2 to 8 nm and did not have surface defects. Scale bar indicates 50 nm.**

**Figure 3:**
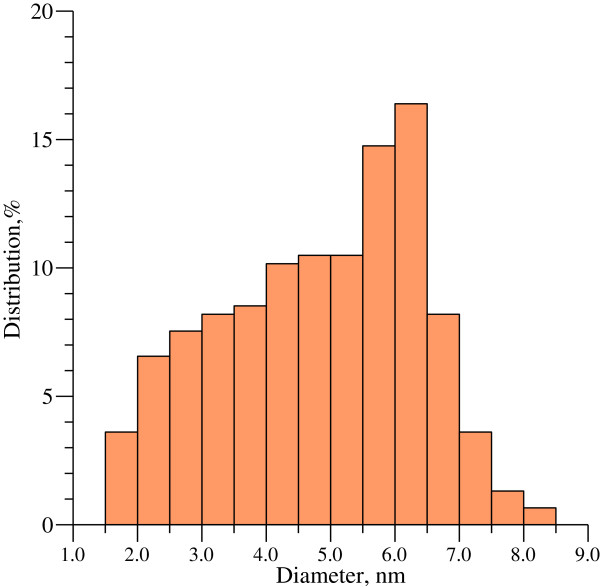
**Particle size distribution histogram determined from the TEM images.** The histogram illustrated number of particles that were in the field of view of the transmission electron microscope. Total number of nanoparticles was 305.

**Figure 4:**
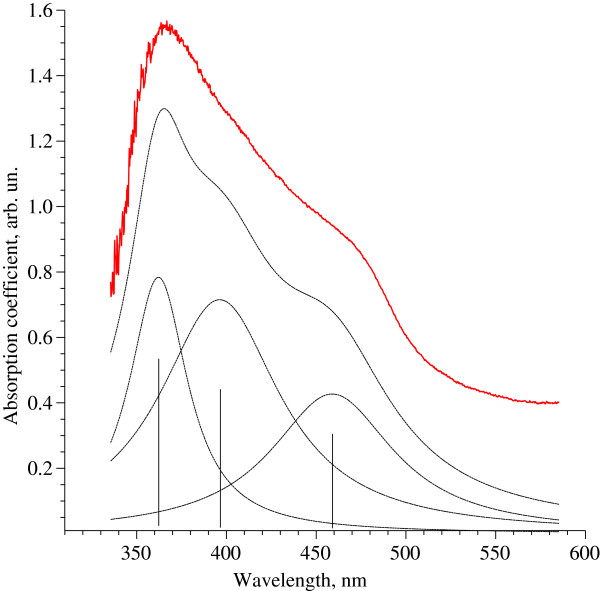
**UV-Visible absorption spectrum of CdS nanoparticles.** Only freshly prepared samples were used for the measurements. Cadmium sulfide quantum dots samples contained 2 ml of 0.025 М CdSO_4_ solution, 30 ml of a hairy root aqueous extract of *L. maroccana* and 500 μl of 0.5 М Na_2_S. A homogeneous clear solution of CdS nanoparticles with bright yellow color was formed after 4 day incubation at 28°C. Absorption spectrum contained three clear peaks corresponded to the wavelengths 362 nm, 398 nm and 464 nm.

Transmission electron microscopy analysis allowed to investigate the shape of produced CdS nanoparticles and their size distribution. For such measurements 0.5 mL of the colloidal solution of CdS nanoparticles was deposited onto carbon coated grids. Typical electron micrographs are represented in Figures 1 and 2.The particle size distribution histogram determined from TEM micrographs is shown in Figure 3. Total number of nanoparticles for particle size histogram is 305 ones per field of view. It was found that the average particle size was 5.5 – 6.9 nm as well as there was a large amount of nanoparticles with a diameter of 2–5 nm. The smallest size of quantum dots corresponded to 7–8.5 nm. In addition according to electron microscopy data, it was established that synthesized nanoparticles were elliptic or spherical in shape and did not have significant surface defects.The absorption spectrum of CdS nanoparticles demonstrated two clear maxima and plangent structure in the interval between these peaks (Figure 4). Total synthesized spectrum is shown in Figure 3 by dashed line. It was revealed that centers of absorption peaks corresponded to the wavelengths 362 nm, 398 nm and 464 nm.

It is known that semiconductor nanocrystals are characterized by energy absorption edge which is shifted toward the absorption band of the CdS macrocrystals in the shortwave region. This "blue" shift is caused by quantum size effects. The energy of band gap for CdS macrocrystals is 2.42 eV that corresponds to the wavelength λ = 512 nm. This "blue" shift indicates the presence of semiconductor nanoparticles in the tested system [20]. Size of the synthesized CdS nanoparticles was estimated from the shift of the absorption band edge in nanoparticles relative to CdS macrocrocryctals by empirical formula [21]


It was established that peak at 362 nm corresponded to the nanoparticles fraction with a diameter 2.5 nm. Maximum at 398 nm corresponded to the particles fraction with a size 3.4 nm, while absorption maximum at 464 nm corresponded to quantum dots with a diameter of 6 nm. Obtained data correlated well with data on particle size distribution in Figure 2. It is worth noting that absorption and luminescence spectra of semiconductor nanoparticles depended on the composition of these nanoparticles, their size and surface features. In particular, it was found that most of the surface defects of nanoparticles, namely, foreign adsorbed atoms or point structure defects can act as potential wells or barriers for holes and electrons. Interaction of the particle surface with an environment and interaction between individual nanoparticles themselves could also affect their optical characteristics. Latter can occur through electronic or resonance energy transfer [22].

**Figure 5:**
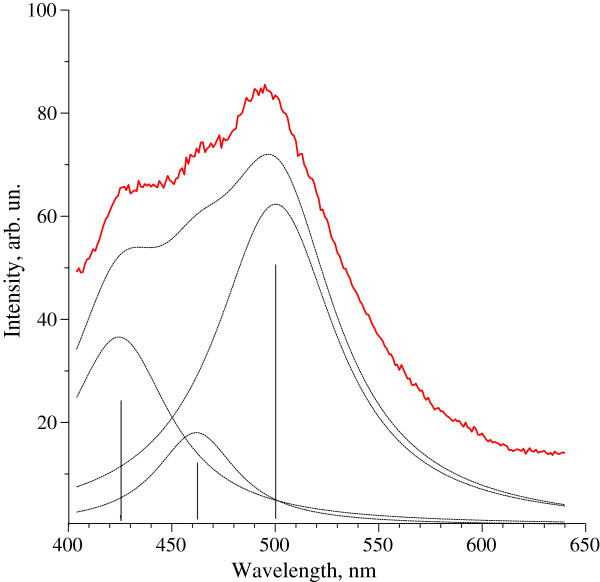
**Luminescence spectrum of CdS nanoparticles.** Freshly prepared samples were used. for the measurements. Cadmium sulfide quantum dots samples contained 2 ml of 0.025 М CdSO4 solution, 30 ml of a hairy root aqueous extract of L. maroccana and 500 μl of 0.5 М Na2S. Inorganic salts were needed as external sources of cadmium and sulfide ions. Luminescence spectrum was measured at excitation λ = 340 nm. Three clear maxima corresponded to the wavelengths 425 nm, 462 nm and 500 nm.

Luminescence spectrum of freshly prepared CdS quantum dots under mercury-vapour lamp excitation λ = 340 nm (Figure 5) was typical for nanodimension CdS [20]. It contained three clear maxima that were overlapped. Total synthesized spectrum is demonstrated in Figure 4 by dashed line. As a result it was revealed that luminescence maxima corresponded to the wavelengths 425 nm, 462 nm and 500 nm. It is believed that at excitation λ = 340 nm (3.65 eV) these luminescent peaks correspond to transitions 1_*se*_ - 1_*sh*_ between dimensional quantization levels in CdS nanoparticles with different diameters. Using previously established relationship between energy of the optical transition 1_*se*_ - 1_*sh*_ and diameter of the CdS nanoparticles [23], we determined that luminescence peaks at 425 nm (2.92 eV), 462 nm (2.68 eV) and 500 nm (2.48 eV) corresponded to transitions 1_*se*_ - 1_*sh*_ in cadmium sulfide nanoparticles with a diameter of 3.8 nm, 5.2 nm and 6.9 nm, respectively. Moreover, the fact that spectrum in the region 460–500 nm was wide enough indicated the presence of a significant amount of CdS nanoparticles with a diameter from 5 to 7 nm in a tested specimen. This size range of synthesized nanoparticles correlated well with obtained TEM data and optical absorption spectra.

**Figure 6:**
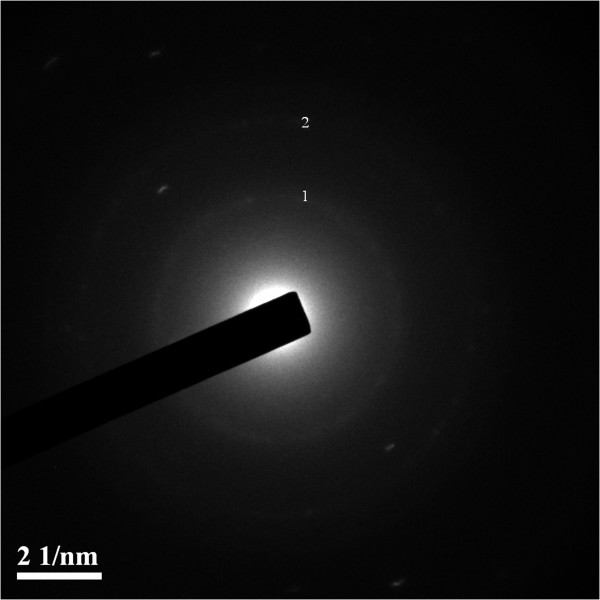
**Electron diffraction pattern of CdS nanoparticles.** The diffraction maxima 1 and 2 corresponded to interplanar distances 0.338 nm and 0.184 nm. Such lattice imaging confirmed that the particles had wurtzite modification.

Using electron diffraction spectroscopy we obtained electron diffraction patterns of cadmium sulfide nanocrystals deposited on carbon coated copper grid (Figure 6). The diffraction maxima 1 and 2 correspond to interplanar distances 0.338 nm, 0.184 nm. This was comparable with previously reported data where the following interplanar distances were complied with CdS nanocrystals structure, indicating polycrystalline wurtzite modification [24]. In our previous research electron diffraction analysis of CdS quantum dots also confirmed wurtzite structure of cadmium sulfide nanocrystals [16]. In addition, in our early investigation [14] it was found that diffraction maxima corresponded to the interplanar distances 0,341 nm, 0,209 nm, and 0,1876 nm which are typical for wurtzite modification of CdS.

Obtained results could also be compared to the data on extracellular biosynthesis of CdS by using the pathogenic fungus *Fusarium oxysporum* where the absorption spectra of CdS nanoparticles had a maximum at 450 nm [25]. Those particles had also a spherical shape and a size from 5 to 20 nm. Furthermore, our previous report demonstrated that a mycelium of the oyster mushroom *Pleurotus ostreatus* was also capable to synthesize eco-friendly and luminescent cadmium sulfide quantum dots [16]. In that case it was observed typical for nanodimension CdS absorption and luminescence spectra as well as the presence of semiconductor nanoparticles with different diameters and we suggested that specific enzymes existing in fungi might be involved in the formation of CdS nanocrystals.

Our previous research [14] showed that maximum luminescence peak of CdS nanocrystals was at 443 nm which was typical for cadmium sulfide nanoparticles synthesized using microorganisms. The stability of obtained quantum dots was investigated for the first time by spectral analysis. It was established that nanoparticles were aggregated, however retained the ability to luminescence for 10 days, 1 and 3 months after a sample preparation [14].

Similar investigation was carried out with the filamentous fungus *Aspergillus versicolor.* The synthesized cadmium sulfide nanoparticles were characterized by spectroscopic and microscopic techniques. The results confirmed the binding of cadmium with sulphur groups of the functionalized mycelia [26]. Formation of 3.0 ± 0.2 nm sized CdS nanoparticles was confirmed by High-resolution transmission electron microscopy (HRTEM) measurements. An increase in adsorption capacity was attributed to cadmium binding affinity of sulfur atoms due to soft acid–base reaction and supported by a − Δ*G* value [26].

It was also demonstrated a simple route for the synthesis of cadmium sulfide nanoparticles by photosynthetic bacteria *Rhodopseudomonas palustris* [27]. The cadmium sulfate solution incubated with *R. palustris* biomass changed to a yellow color from 48 h onward indicating the formation of cadmium sulfide nanoparticles. The purified solution yielded the maximum absorbance peak at 425 nm due to CdS particles in the quantum size regime. TEM analysis of the samples showed a uniform distribution of nanoparticles with an average size of 8.01 ± 0.25 nm [27]. Biosynthetic ability of this bacterium was found to be strain-dependent.

In present study an extract of hairy root of plant *L. maroccana* was used for the first time for the biosynthesis of semiconductor nanoparticles. We focused on hairy root culture because of its fast growth *in vitro* under controlled conditions, possibility to obtain for the short period of time a large increase in root biomass enriched in secondary metabolites that are involved in the CdS quantum dots biosynthesis process. Probably, these secondary metabolites might be involved in the process of the reduction of the sulfate groups. But exact extracellular mechanism of CdS formation with the use of plant systems still remains unclear and requires further detailed investigation.

## Conclusions

CdS nanocrystals synthesized by us using plant extract had typical for quantum dots optical properties such as specific absorption and luminescent spectra, an ellipsoid or a spherical shape as well as the particle size distribution ranging from 1.5 nm to 8.5 nm. Because the size of nanoparticles is a defining feature in their applications the ability to control the dimensions of synthesized CdS nanoparticles through variations of the biosynthesis conditions draws a great attention. Produced by described "green" approach CdS nanoparticles are promising for use in cell biology, particularly as a new generation of fluorophores.

## Authors’ information

M.N. Borovaya (PhD student), A.P. Naumenko (PhD), N.A. Matvieieva (PhD),Ya.B. Blume (Prof.), A.I. Yemets (Prof.).
